# Strength Improvements of Different 10-Week Multicomponent Exercise Programs in Elderly Women

**DOI:** 10.3389/fpubh.2020.00130

**Published:** 2020-05-12

**Authors:** José María González-Ravé, Rubén Cuéllar-Cañadilla, Teresa García-Pastor, Daniel Juárez Santos-García

**Affiliations:** ^1^Department of Physical Activity and Sport Sciences, University of Castilla la Mancha, Toledo, Spain; ^2^Department of Sport Sciences, University of Camilo Jose Cela, Madrid, Spain

**Keywords:** strength, training, female, physical programs, jumps

## Abstract

The purpose of this study was to analyze and compare the effects of various muscle strength sessions performed during 10 weeks physical conditioning programs and 4 weeks of detraining on the physical conditioning, body composition and hematology of elderly women. 48 women were randomly distributed in four groups: a physical conditioning group (PCG; *n* = 12) with two sessions per week; a PCG with an extra-session focus on hypertrophy (PCGH; *n* = 12), a PCG with an extra-session in a shallow pool (PCGP; *n* = 12); and a control group (CG; *n* = 12). PCGH achieved a significant increase in Abalakov. Significant differences between the CG and the other groups were found in Countermovement Jump. There were significant improvements in the chair-stand test in the PCG and PCGH. The training programs suspected improvements in HDL, LDL, baseline glucose and glycated hemoglobin. In conclusion, a training program with an extra-session focus on hypertrophy achieved a greater improvement in strength in elderly women.

## Introduction

The physiological and anatomical characteristics of elderly adults deteriorate over the years. Their functional capacity decreases and changes occur at the respiratory, cardiovascular, renal, nervous and muscle-skeletal level (1). In women, especially after menopause, various physical abilities (strength, flexibility, equilibrium, resistance, and agility) follow a natural evolution of loss with age (2).

Over the last 10 years, there have been numerous and varied physical conditioning programs aimed at adults older than 60 years. These programs have sought to improve physical characteristics and body composition, optimizing and improving the quality of life of these individuals (3–6).

One of the most common programs that has proliferated it's self in social/cultural centers and in municipal sports programs is the general physical conditioning or multicomponent program This sort of exercise plan has had positive effects on the health in women older than 60 years (6, 7).

However, most scientific literature has focused on strength training in elderly adults. Most studies that are focused on increasing the muscle strength and functional strength of elderly adults using hypertrophy or neural adaptation methods were implemented in a period varying between 6 and 16 weeks, with a weekly frequency of two to three sessions, obtaining strength gains in upper and lower limbs (8–13).

Additionally, all of these programs caused changes, which if not performed periodically can lead to losses in strength, functional performance and body composition after the end of the training periods (12, 14). The clinical relevance of the neuromuscular adaptations induced by strength training is its impact on the daily living activities, especially when the strength training performed for several weeks led to greater improvements in strength in the arms and legs.

Therefore, Considering the importance of physical exercise in women older than 60 years, particularly strength training, it is essential to continue analyzing different physical conditioning programs in order to determine the best protocols for improving physical fitness in this population.

The aim of this study was to analyze and compare the effects of various muscle strength sessions implemented in 10-week physical conditioning programs, with a 4 weeks detraining, on the physical conditioning, body composition and hematology of elderly women.

## Materials and Methods

### Design

In an interventional controlled trial, a general conditioning program alone, the same program with a focus on hypertrophy, or the same program with an extra pool session was used to analyze the effects of different training sessions and deconditioning after completing the program in elderly women. The program implemented lasted 10 weeks, followed by a 4 weeks detraining period.

### Subjects

Forty-eight women (mean age, 65.7, *s* = 4.5 years; mean weight, 75.3, *s* = 11.1 kg) voluntarily participated in this study. The sample size was calculated by estimating a large effect size, α of 0.05, and power of 0.8. The sample was divided into four groups and four measurement moments were established (pre-post 1 (after the training program), post 2 and post 3 (two and 4 weeks after finishing the training program). Participants had performed in 3–5 years of controlled physical exercise.

Participants were randomly selected from people involved in the senior activity program of the Community Center of Madridejos (Toledo, Spain). All tests and training programs were carried out at the Sport city center which belongs to the Municipality of Madridejos. The participants were assessed between February and June of 2016. They were checked thoroughly during all medical procedures by medical staff (doctor and nurse). To be included in the study participants had to be aged 60 and 80 years old, and live within 50 km of a metropolitan area.

The inclusion criteria were the following: women between the ages of 60 and 80 years participating for more than 3 years in elderly physical exercise programs in the Municipality of Madridejos (Toledo, Spain) The exclusion criteria were a history of cardiac, respiratory or joint movement problems, problems with dizziness during exertion, propensity for infections, use of drugs that could endanger the participant during the implementation of the program and participation in a physical conditioning program aimed at developing strength in the past 6 months.

A parallel group design was performed. The participants were randomly distributed to four groups: a general physical conditioning group (PCG; *n* = 12), a general physical conditioning group with hypertrophy development (PCGH; *n* = 12), a general physical conditioning group with strength development in a swimming pool (PCGP; *n* = 12) and a control group (CG; *n* = 12).

The participants were informed of the benefits and risks prior to signing the informed consent document to participate in the research. The study was approved by the Clinical Research Ethics Committee of the University of Castilla-La Mancha and conducted in accordance with the Helsinki Declaration.

### Procedures

#### Training Program

The training programs were conducted for 10 weeks from February to April, 2016. Previously, all groups had performed two familiarization sessions with the assessment tests, and the training groups were also familiarized with the exercises to be performed. All study groups had several training elements in common (Table 1). The participants were asked not to participate in other physical activities during the study period.

**Table 1:** Exercise program performed by training groups.

**Frequency**	**Intensity**	**Time**	**Type**
Once a week.	60–70% Estimated heart rate (HR), combined with 70–80% (speech test).	40 min	Mean aerobic (cardiovascular exercises with musical base) combined with intermittent exertions.
Once a week.	60–70% 1RM; Character of exertion (12–15 of 20 repetitions) with moderate execution speed.	8–10 exercises × 3 × 12–15/30”	Strength-resistance (use of auxiliary material and calisthenics with the body itself.
Twice a week.	Joint range of movement (ROM) (Progressive reach of the maximum).	(5 exercises × 1–3 × 20”/10”)/ session	Flexibility (Joint ROM improvement). Stretching.
Once a week.	Progression in the difficulty level (no. of supports, equilibrium base, distribution of supports).	5 exercises × 2'/30”	Static and dynamic equilibrium.
Once a week.	Progression in the difficulty level (from hand-eye coordination to foot-eye coordination; from intraindividual to interindividual coordination; stable environments to unstable environments).	3 exercises × 3'/30”	Coordination and agility.

The training was differentiated as follows:

- PCG: performed the previous program exclusively.
- PCGH: the extra session performed per week can be seen in (Table 2). In the familiarization sessions, we determined the work intensity, calculating the 12 repetition maximum (RM) of the exercises to be performed during the program (except in the abdominal and dorsal-lumbar extension exercises). We used variable resistance machines. Work was performed with the following exercise circuit, according to (Table 2).
  ○ Circuit 1: 1-Leg extension in pulley. 2- Rectus abdominis (Extended leg crunch) (25 reps). 3- Low pulley row. 4- Abductors seated on machine (Gluteus maximus). 5- Dorsal-lumbar extension in lying prone position (25 reps). 6- Alternating biceps curl with supination (seated). 7- Lifting the heels in the machine. 8- Alternating frontal liftings with low pulley.
  ○ Circuit 2: 1- Leg curl lying down (hamstrings). 2- Oblique abdominals (25 reps). 3- Bench press in machine (Pectoralis major). 4- Abductors seated on machine. 5- Triceps extension in high pulley. 6- Unilateral dorsal-lumbar extension in lying prone position (25 reps). 7- Leg press inclined. 8. Pulldown with pulley to chest.
  ○ Circuit 3: 1-Pull over with high pulley (latissimus dorsi). 2- Semi-squat in multipower. 3- Anterior deltoids in machine (shoulder press). 4- Gluteus medius (hip abduction standing in machine). 5- Abdominal crunches with legs (25 reps). 6- Butterfly chest contractions. 7- Unilateral dorsal-lumbar extension in lying prone position (25 reps). 8- Lateral lunges with dumbbells.
- PCGP: performed a training similar to that of the previous group in terms of muscles involved but different in terms of intensity and speed of execution (Table 3). This session was performed in a heated swimming pool and with the use of various auxiliary materials (boards, pull-buoys, pool noodles, and paddles). A musical base was employed to increase the participants' motivation.

**Table 2:** Programming of the specific training group with an extra strength session (PCGH).

**Week**	**Volume**	**Intensity**	**Exercises**
1	8 exercises × (2 × 12/1')/3'	•70% 1RM	CIRCUIT 1
2	8 exercises × (2 × 12/1')/3'	•70% 1RM	CIRCUIT 2
3	8 exercises × (2 × 12/1')/3'	•70% 1RM	CIRCUIT 3
4	8 exercises × (3 × 12/1')/3'	•70% 1RM	CIRCUIT 1
5	8 exercises × (3 × 12/1')/3'	•70% 1RM	CIRCUIT 2
6	8 exercises × (3 × 12/1')/3'	•70% 1RM	CIRCUIT 3
7	8 exercises × (3 × 8/2')/3'	•80% 1RM.	CIRCUIT 1
8	8 exercises × (3 × 8/2')/3'	•80% 1RM	CIRCUIT 2
9	8 exercises × (3 × 8/1'30”)/3'	•80% 1RM	CIRCUIT 3
10	8 exercises × (3 × 8/1'30”)/3'	•80% 1RM	CIRCUIT 1

*1RM, 1 repetition maximum*.

**Table 3:** Factors for the programming of the specific block in the PCGP training group.

**Week**	**Volume**	**Intensity**	**Exercises**
1	8 exercises × (2 × 20/30”)/2' (active rest).	•RPE (Borg): 7 •Bpm: 120	CIRCUIT 1
2	8 exercises × (2 × 20/30”)/2' (active rest).	•RPE (Borg): 7 •Bpm: 120	CIRCUIT 2
3	8 exercises × (2 × 20/30”)/2' (active rest).	•RPE (Borg): 7 •Bpm: 120	CIRCUIT 3
4	8 exercises × (3 × 20/30”)/2' (active rest).	•RPE (Borg): 7 •Bpm: 120	CIRCUIT 1
5	8 exercises × (3 × 20/30”)/2' (active rest).	•RPE (Borg): 7 •Bpm: 120	CIRCUIT 2
6	8 exercises × (3 × 20/30”)/2' (active rest).	•RPE (Borg): 7 •Bpm: 120	CIRCUIT 3
7	8 exercises × (3 × 20/30”)/2' (active rest).	•RPE (Borg): 8 •Bpm: 130	CIRCUIT 1
8	8 exercises × (3 × 20/30”)/2' (active rest).	•RPE (Borg): 8 •Bpm: 130	CIRCUIT 2
9	8 exercises × (3 × 20/30”)/2' (active rest).	•RPE (Borg): 8 •Bpm: 130	CIRCUIT 3
10	8 exercises × (3 × 20/30”)/2' (active rest).	•RPE (Borg): 8 •Bpm: 130	CIRCUIT 1

*Bpm, beats per minute; RPE, Rating of Perceived Exertion*.

The exercise circuits performed were as follows:

Circuit 1

1- Jumping Jack (opening and closing of the legs) with extension/flexion of the elbow. 2- Rectus abdominis (V with legs bent). 3- Traction with board in the water. 4- Knee extensions with lateral inclination (Lateral kicking). 5- Ventral to dorsal flotation. 6- Bicep supination with paddles. 7- Multiple tip-toe hops. 8- Shoulder adduction with pull-buoys.

Circuit 2

1- Femoral (heel to buttock). 2- Oblique abdominals (unilateral inclination with elbow extension). 3- Pushing with boards. 4- Pendulum to 1 leg with pool noodle (hip adduction). 5- Elbow extensions with paddles. 6- Ventral to dorsal flotation. 7- Alternating knee extension (in suspension). 8- Frontal shoulder adduction with pull-buoys.

Circuit 3

1- Tractions with pull-buoys. 2- Hops with semi-squat. 3- Deltoids with board (vertical elbow extension). 4- Hip extensions. 5- Rectus abdominis. 6- Unilateral push with elbow extension and with pull-buoys. 7- Lateral turns using the elbows in flexion. 8- Lateral lunge with elbow extension and board facing forward.

After completing the training programs (POST1), the assessment tests were repeated 2 (POST2) and 4 (POST3) weeks later (except for the blood analysis, which was performed only once [POST1] after the intervention).

### Testing procedures

Prior to applying the training programs, the following measurements were performed, scheduling each group on a different day:

- Blood analysis, recording the cholesterol (total, HDL and LDL), hemoglobin (Hb), HB (HbA1c), hematocrit (HCT) and glucose.
- Body composition analysis through bioimpedance (Inbody 230, Biospace Co. Ltd., Seoul, South Korea).
- Countermovement jump test (CMJ) and Abalakov (ABK) using the Optojump contact barriers (Microgate, Italy), performing two attempts of each jump, separated by 2 min, and selecting the highest jump.
- Test for standing and sitting in a chair the highest number of times possible in 30 s. This test is part of the battery of tests known as the Senior Fitness Test (STF) (15). The test consists of sitting down and standing up as many times as possible in 30 s, without using the hands. This test was performed using a chair without armrests, with a height of 40 cm. The chair was placed against a wall to prevent it from moving during the implementation of the test. The test began with the individual seated in the chair, with the back straight and supported on the backrest, the feet parallel and placed on the floor in a straight line with the knees to maintain balance during the test. The arms are crossed at the chest.
- Biceps curl test with 2.5 kg for 30 s (15). To start the test, the individual must sit down in a chair without armrests with the back straight and the feet completely placed on the floor. The participants took the dumbbell with their dominant hand and placed it perpendicular to the floor, with their elbow against the body and the palm of the hand upwards. The participants lifted the dumbbell to the shoulder and then returned it to the starting position, performing as many repetitions as possible within 30 s.
- The 6-min walking test covering the longest distance possible, covering a rectangular circuit (29 × 0.5 m). The short walking test has been shown to correlate well with cardiopulmonary resistance in elderly adults with differing health conditions (16, 17).

### Statistical Analyses

The SPSS v.22 program was employed for the data analysis. The normality of the variables was analyzed with the Shapiro-Wilk test. Homoscedasticity was checked with Levene's test. To analyze the differences between groups for each of the variables, we applied an ANOVA of repeated measures (group × moment) with a Bonferroni *post hoc* test. Statistical significance was established at *p* < 0.05. We calculated the size of the effect using the partial eta squared (η^2^) in the ANOVA analysis and through Cohen's d in the comparisons by pairs.

## Results

The results of all parameters are showed in (Tables 4–11). There were no significant differences in the changes between the groups or between the various assessments performed for the body weight, body fat percentage and muscle mass variables.

**Table 4:** Results of Abalakov jump (cm) test (mean ± SD).

	**CG (*n* = 12)**	**PCG (*n* = 12)**	**PCGH (*n* = 12)**	**PCGP (*n* = 12)**
Pre-training	6.71 ± 3.73	10.18 ± 3.99	8.53 ± 1.93	7.17 ± 3.25
Post-training1	7.22 ± 3.88	10.68 ± 3.23	10.20 ± 2.39^**^	7.87 ± 2.77
Post-training2	6.05 ± 3.19	9.97 ± 3.54	10.83 ± 3.34^**^	8.42 ± 2.79
Post-training3	6.55 ± 3.46	9.78 ± 3.75	10.15 ± 3.25^**^	8.07 ± 3.03

** *Difference (p < 0.01) with pre-training*.

**Table 5:** Results of CMJ (cm) test (mean ± SD).

	**CG (*n* = 12)**	**PCG (*n* = 12)**	**PCGH (*n* = 12)**	**PCGP (*n* = 12)**
Pre-training	6.51 ± 3.03	8.76 ± 2.86	8.08 ± 1.91	6.16 ± 2.57
Post-training1	5.61 ± 2.87	9.17 ± 3.34^#^	8.85 ± 1.66	7.07 ± 2.82
Post-training2	5.49 ± 2.83	9.18 ± 3.38^#^	9.87 ± 2.15^##^^**^^&^	7.29 ± 2.29^*^
Post-training3	5.87 ± 3.02	9.38 ± 2.89^#^	9.51 ± 2.56^#^^*^	7.47 ± 2.15^*^

# *Difference (p < 0.05) with CG*.

## *Difference (p < 0.01) with CG*.

* *Difference (p < 0.05) with pre-training*.

** *Difference (p < 0.01) with pre-training*.

& *Difference (p < 0.05) with post-training1*.

**Table 6:** Results of Biceps curl in 30 s (repetitions) test (mean ± SD).

	**CG (*n* = 12)**	**PCG (*n* = 12)**	**PCGH (*n* = 12)**	**PCGP (*n* = 12)**
Pre-training	10.37 ± 3.25	13.92 ± 2.60	13.25 ± 3.05	12.17 ± 2.92
Post-training1	12.62 ± 3.70	15.77 ± 2.59	16.17 ± 1.75^**^	14.25 ± 3.05^*^
Post-training2	14.00 ± 3.62^**^	16.23 ± 2.65^**^	16.58 ± 1.83^**^	14.92 ± 2.43^**^
Post-training3	14.12 ± 3.48^**^	16.92 ± 2.22^**^	16.25 ± 1.76^**^	14.83 ± 2.25^**^

* *Difference (p < 0.05) with pre-training*.

** *Difference (p < 0.01) with pre-training*.

**Table 7:** Results of Stand Chair (repetitions) test (mean ± SD).

	**CG (*n* = 12)**	**PCG (*n* = 12)**	**PCGH (*n* = 12)**	**PCGP (*n* = 12)**
Pre-training	14.00 ± 1.41	15.85 ± 2.15	16.33 ± 1.56	14.25 ± 2.14
Post-training1	14.12 ± 2.85	17.08 ± 2.36^#^^*^	18.00 ± 1.71^##^^**^	14.67 ± 2.50^##^^&^
Post-training2	13.00 ± 1.93	16.92 ± 2.22^##^	17.08 ± 1.93^##^	14.75 ± 2.60
Post-training3	13.25 ± 2.05	17.46 ± 2.96^##^^@^^**^	17.17 ± 1.40^##^^@^	14.33 ± 2.42

# *Difference (p < 0.05) with CG*.

## *Difference (p < 0.01) with CG*.

& *Difference (p < 0.01) with PCGH*.

@ *Difference (p < 0.05) with PCGP*.

* *Difference (p < 0.05) with pre-training*.

** *Difference (p < 0.01) with pre-training*.

**Table 8:** Results of 6-min walking (m) test (mean ± SD).

	**CG (*n* = 12)**	**PCG (*n* = 12)**	**PCGH (*n* = 12)**	**PCGP (*n* = 12)**
Pre-training	474.37 ± 55.90	533.08 ± 60.30	501.67 ± 87.32	484.58 ± 61.62
Post-training1	465.62 ± 65.16	541.92 ± 28.25	537.92 ± 33.13	508.33 ± 38.45
Post-training2	450.00 ± 56.38	540.00 ± 31.22	525.83 ± 31.83	514.58 ± 29.88
Post-training3	468.75 ± 50.20	541.15 ± 35.72	525.83 ± 35.73	513.75 ± 39.03

**Table 9:** Results of body fat (%) and muscle mass (kg) analysis (mean ± SD).

	**GC (*n* = 12)**	**PCG (*n* = 12)**	**PCGH (*n* = 12)**	**PCGP (*n* = 12)**
**Body fat (%)**				
Pre-training	46.44 ± 7.42	45.09 ± 4.19	45.82 ± 4.15	45.49 ± 3.00
Post-training1	47.47 ± 7.36	45.02 ± 4.20	45.97 ± 4.54	46.02 ± 3.32
Post-training2	46.85 ± 6.91	45.12 ± 3.67	45.52 ± 4.82	45.70 ± 3.20
Post-training3	47.34 ± 7.38	44.48 ± 4.49	45.68 ± 4.56	45.59 ± 3.44
**Muscle mass (Kg)**				
Pre-training	22.99 ± 2.76	22.33 ± 2.87	22.26 ± 3.22	21.37 ± 3.05
Post-training1	21.70 ± 2.60	22.40 ± 2.69	22.01 ± 3.17	21.19 ± 3.56
Post-training2	22.00 ± 2.82	22.27 ± 2.52	22.00 ± 3.11	21.31 ± 3.21
Post-training3	21.86 ± 2.45	22.71 ± 2.98	21.96 ± 3.35	21.37 ± 3.08

**Table 10:** Results of total cholesterol. LDL and HDL (mg/dl) analysis (mean ± SD).

	**CG (*n* = 12)**	**PCG (*n* = 12)**	**PCGH (*n* = 12)**	**PCGP (*n* = 12)**
**Total cholesterol (mg/dl)**				
Pre-training	194.25 ± 22.82	201.23 ± 23.53	208.58 ± 27.63	203.00 ± 22.37
Post-training1	207.25 ± 11.45^**^	200.31 ± 11.87	203.25 ± 25.37	202.13 ± 17.62
**LDL (mg/dl)**				
Pre-training	120.49 ± 25.01	117.41 ± 14.99	128.36 ± 18.92	113.94 ± 13.04
Post-training1	123.19 ± 17.77	114.49 ± 10.37	123.36 ± 16.83^*^	110.28 ± 13.32
**HDL (mg/dl)**				
Pre-training	56.50 ± 5.24	53.92 ± 8.20	51.75 ± 5.59	54.25 ± 4.97
Post-training1	58.38 ± 7.48	56.77 ± 5.57^*^	54.33 ± 4.07	56.17 ± 6.10

* *Difference (p < 0.05) with pre-training*.

** *Difference (p < 0.01) with pre-training*.

**Table 11:** Results of Hemoglobin (g/dl), Hematocrit (%), Glucose (mg/dl) and Glycated Hemoglobin (%) analysis (mean ± SD).

	**CG (*n* = 12)**	**PCG (*n* = 12)**	**PCGH (*n* = 12)**	**PCGP (*n* = 12)**
**Hemoglobin (g/dl)**				
Pre-training	13.94 ± 0.81	13.97 ± 0.78	13.87 ± 0.66	13.92 ± 0.67
Post-training1	13.99 ± 0.79	14.25 ± 0.69	13.71 ± 0.69	14.07 ± 0.71
**Hematocrit (%)**				
Pre-training	40.06 ± 2.90	41.00 ± 3.38	40.30 ± 2.27	40.47 ± 2.39
Post-training1	39.87 ± 2.81	41.28 ± 2.97	39.72 ± 2.47	41.02 ± 2.30
**Glucose (mg/dl)**				
Pre-training	97.63 ± 10.07	98.85 ± 7.64	99.08 ± 9.59	102.17 ± 9.65
Post-training1	98.88 ± 12.26	97.77 ± 8.68	91.42 ± 7.24^**^	99.75 ± 10.70
**Glycated hemoglobin (%)**				
Pre-training	5.54 ± 0.29	5.58 ± 0.32	5.61 ± 0.28	5.78 ± 0.25
Post-training1	5.70 ± 0.29^*^	5.46 ± 0.29^*^	5.47 ± 0.24^*^	5.68 ± 0.35

* *Difference (p < 0.05) with pre-training*.

** *Difference (p < 0.01) with pre-training*.

*CG, control group; PCG, general physical conditioning group; PCGH, general physical conditioning group (hypertrophy); PCGP, general physical conditioning group (pool)*.

In terms of the variables obtained with the blood tests, we saw differences between the resulting changes between the groups. In the cholesterol-related variables, these differences were primarily observed in total cholesterol (*p* = 0.02; η = 0.2), mainly by the difference in LDL (n.s.; η = 0.1). In the Hb, we mainly saw a difference in the glycated Hb (p=0.005; η = 0.3; for the Hb n.s.; η = 0.08). There were no significant differences in the hematocrit (n.s.; η = 0.06). In terms of glucose, the PCGH showed a significant reduction (*p* = 0.001; *d* = 1.1) after the 10 weeks of training.

The results of the jump height in ABK showed no significant differences among groups or when analyzing only the 10 weeks of training or when considering the entire study period. However, we observed that the PCGH achieved an increase (*p* < 0.01, *d* = 0.7) with the training program, without changes among Post training 1, 2, and 3, although these changes are not significant (ns; *d* = 0.2), the values are still greater than the PRE value (*p* < 0.01, *d* = 0.7, for the PRE-POST2 difference, *d* = 0.5, for the PRE-POST3 difference). Therefore, no significant differences between-group were found on the ABK measure, but there was a significant within-group difference in the PCGH group.

The results of the ANOVA showed that there were differences (*p* < 0.01; η = 0.20) in the changes produced among all the groups in jump height in CMJ, although these differences were not observed right after the completion of the program but rather subsequently. Differences were found between the CG and the other groups (*p* < 0.01; η = 0.61, with the PCGH; *p* < 0.01; η = 0.25, with the PCGP; *p* < 0.05; η = 0.12, with the PCG). Both the PCGH and PCGP obtained higher values in POST2 and POST3 compared with the PRE value (*p* < 0.01; *d* = 0.8, *d* = 0.5, in the PCGH between PRE and POST2 and between PRE and POST3, respectively; *p* < 0.01; *d* = 0.5, *d* = 0.6, in the PCGP between PRE and POST2 and between PRE and POST3, respectively).

In the number of repetitions in biceps curl within 30 s, no significant differences were found in the changes between groups.

Although we did not find significant differences in the changes experienced in the chair standing repetitions between the groups, both the PCG (*p* < 0.01, *d* = 0.5) and PCGH (*p* < 0.01, *d* = 1) showed an increase after the training program.

Similarly, we found no significant differences, either after the 10 weeks of training or in the entire study period, between the groups in the distance traveled in the 6-minute walking test, although the 3 training groups increased their distance while the control group decreased their distance. The most relevant increase was that of the PCGH (*d* = 1.1) followed by the PCGP (*d* = 0.6).

## Discussion

The aim of this study was to analyze and compare the effects of various muscle strength sessions implemented in 10-week physical conditioning programs and 4 weeks of detraining on the physical condition, body composition and hematology of elderly women. Recent studies have shown that elderly women improved their physical fitness following multicomponent exercise (6, 7). Toto et al. (6) analyzed the effects of a 10-week multicomponent program and they observed a improving physical performance and activities of daily living. Kang et al. (7) affirmed that multicomponent training programs that consist of “balance, strengthening, and stretching exercises are a relevant intervention for the improvement of the level of physical fitness of older women.”

No significant differences between programs were found in body composition. A possible explanation for these results is the fact that although a number of the subjects had some significant improvements in physical fitness, they did not decrease fat mass. This fact suggests that changes in body composition should not be the only indicator of the benefits of exercise in overweight postmenopausal women, as shown by Myette-Côté et al. (18) in their study. Halverstadt et al. (19) found no changes in body composition after 24 weeks of endurance exercise training. In addition, Amarante et al. (20) suggested that a 12-week resistance-training program (3 times a week) improved muscle strength without changes in body composition in elderly women under dietary intake maintenance.

Differences between the groups were found in some of the blood parameters after 10 weeks of the training program, which agrees with Ribeiro et al. (21). Halverstadt et al. (19) observed improvements in plasma lipoprotein and lipid profile with independence of diet and baseline levels after 24 weeks of endurance exercise training. Tomeleri et al. (22) performed a resistance training program consisting of 8 whole-body exercises for 3 sets of 10–15 repetition maximum (RM) performed three times per week with obese elderly women. The program improved inflammatory levels and the lipid and glycemic profiles. Our results agree with those of Martins et al. (23) who concluded that “training programs produced significant benefits in metabolic health indicators of sedentary elderly women and men.”

There were no significant differences between the groups after 10 weeks of the physical conditioning program and 4 weeks of detraining in the jump height in ABK. However, the PCGH achieved an increase (*p* < 0.01, *d* = 0.7) between PRETEST and POST1 and between POST1 and POST2. After 4 weeks of training, a significant reduction was produced in POST3. These results agree with those of Delshad et al. (9) who found decreases after 4 weeks of detraining in 50-year-old women. According to the American College of Sports Medicine reports, “strength training is important for improving quality of life and physical function in older adults.” Raj et al. (24) obtained improvements in the jump with a strength training program at 75% of 1RM for 16 weeks of training in older adults. The results of this study also agree with those of González-Ravé et al. (25) who observed improvements in the vertical jump after 16 weeks of strength training in elderly adults. The results of the CMJ confirm how significant improvements were produced in the jump in the PCGH and PCGP between PRETEST and POST2. Differences were also observed between the experimental groups and the control group.

In terms of performance in muscle resistance in the arms and legs, there were no differences in the 30-s biceps curl test between the groups after the training, although the experimental groups showed improvements on the number of repetitions with the various training programs. Amarante et al. (20) obtained similar results after a 12-week resistance-training program (3 times a week) in improving arm muscle strength, without altering the body composition in elderly women. Similar results were found by Daly et al. (8) after arm strength training for 6 weeks. The chair stand results in this study showed significant improvements in the PCG and PCGH. Jones et al. (26) concluded that the 30-s chair stand test was a good indicator of lower body strength in active older adults. Similar results were found by Taguchi et al. (5) but in elderly adults with a mean age of 84 years.

Finally, although the improvements observed in the 6-min walking test were not significant, the groups who performed the training improved their distance traveled in this test. The improvements in cardiovascular resistance in this type of individual can be greater than those observed in this study, given that in our study 40 min a week were dedicated specifically to improving the cardiovascular system, an amount of exercise lower than that recommended by the American College of Sport Medicine (27). This can be observed in other studies, such as the one by Hallage et al. (28), where weekly exercise of more than 150 min aimed at cardiovascular resistance was performed. The study obtained significant improvements after 12 weeks of training, although these improvements decreased significantly after 1 month of detraining.

Finally, the main finding of our study was that physically active older women, at least within in the PCGH, improved their muscle power, endurance and local muscular endurance without improvements in body composition and blood tests.

A particular strength of our study lies in the possibility to compare different conditioning programs for elderly women and the follow-up period that allowed us to measure the maintenance of the changes after the training sessions. However, several limitations should be taken into consideration. The small sample size of the study, specifically the control group with fewer participants than the other groups, or the lack of randomization of the sample did not allow us to show more significant results.

In accordance with González-Ravé et al. (25), any training proposal required to put on special attention and individual experimentation in elderly people. A well-planned multicomponent training program does not entail an additional overload in the development of skeletal muscle in elderly women, as evidenced by an adaptive response without acute fatigue or overtraining, and by biochemical parameters. This is an important finding due to the demonstrated benefits of strength and power improvement for the function and quality of life of elderly people.

## Conclusions

After 10 weeks of training with various conditioning programs for elderly women than 60 years, we observed that the program with an extra strength training session aimed at muscle mass gains resulted in greater improvements in strength in the arms and legs.

The multicomponent exercise programs supposed improvements in various hematological variables (HDL cholesterol, LDL cholesterol, baseline glucose and glycated hemoglobin).

The 4 week detraining period resulted in no significant changes in body composition, physical condition and hematological variables compared with post-training levels. Due to the changes observed after the extra strength training session, in future studies, it would be beneficial to deepen the intensity, frequency, or to analyze the effects of a longer detraining period on this population.

## Data Availability Statement

The datasets generated for this study are available on request to the corresponding author.

## Ethics Statement

The study was approved by the Clinical Research Ethics Committee of the University of Castilla-La Mancha and conducted in accordance with the Helsinki Declaration. The patients/participants provided their written informed consent to participate in this study.

## Author Contributions

RC-C, JG-R, TG-P, and DS-G conceptualized, designed and performed the experiments and analyzed and interpreted the data. RC-C, JG-R, TG-P, and DS-G edited and critically reviewed the manuscript. JG-R and DS-G wrote the manuscript.

## Conflict of Interest

The authors declare that the research was conducted in the absence of any commercial or financial relationships that could be construed as a potential conflict of interest.
